# Interactivity, Quality, and Content of Websites Promoting Health Behaviors During Infancy: 6-Year Update of the Systematic Assessment

**DOI:** 10.2196/38641

**Published:** 2022-10-07

**Authors:** Danielle Jawad, Heilok Cheng, Li Ming Wen, Chris Rissel, Louise Baur, Seema Mihrshahi, Sarah Taki

**Affiliations:** 1 Sydney School of Public Health Faculty of Medicine and Health The University of Sydney Sydney Australia; 2 Health Promotion Unit Population Health Research & Evaluation Hub Sydney Local Health District Sydney Australia; 3 National Health and Medical Research Council Centre of Research Excellence in the Early Prevention of Obesity in Childhood - Translate The University of Sydney Sydney Australia; 4 Susan Wakil School of Nursing and Midwifery Faculty of Medicine and Health The University of Sydney Sydney Australia; 5 Sydney Institute for Women, Children and their Families Sydney Local Health District Sydney Australia; 6 College of Medicine and Public Health Rural and Remote Health South Australia and Northern Territory Flinders University Darwin Australia; 7 Specialty of Child and Adolescent Health Sydney Medical School The University of Sydney Sydney Australia; 8 Department of Health Sciences Faculty of Medicine, Health and Human Sciences Macquarie University Sydney Australia

**Keywords:** breastfeeding, bottle feeding, websites, web-based platform, infant food, readability, accuracy, consumer, health information, interactivity, solid food, quality, grading, comprehensibility, infant, baby, babies, feeding, food, eating, nutrition, health behavior, web-based information, health website, sleep, screen time, rating

## Abstract

**Background:**

As of 2021, 89% of the Australian population are active internet users. Although the internet is widely used, there are concerns about the quality, accuracy, and credibility of health-related websites. A 2015 systematic assessment of infant feeding websites and apps available in Australia found that 61% of websites were of poor quality and readability, with minimal coverage of infant feeding topics and lack of author credibility.

**Objective:**

We aimed to systematically assess the quality, interactivity, readability, and comprehensibility of information targeting infant health behaviors on websites globally and provide an update of the 2015 systematic assessment.

**Methods:**

Keywords related to infant milk feeding behaviors, solid feeding behaviors, active play, screen time, and sleep were used to identify websites targeting infant health behaviors on the Google search engine on Safari. The websites were assessed by a subset of the authors using predetermined criteria between July 2021 and February 2022 and assessed for information content based on the Australian Infant Feeding Guidelines and National Physical Activity Recommendations. The Suitability Assessment of Materials, Quality Component Scoring System, the Health-Related Website Evaluation Form, and the adherence to the Health on the Net code were used to evaluate the suitability and quality of information. Readability was assessed using 3 web-based readability tools.

**Results:**

Of the 450 websites screened, 66 were included based on the selection criteria and evaluated. Overall, the quality of websites was mostly adequate. Media-related sources, nongovernmental organizations, hospitals, and privately owned websites had the highest median quality scores, whereas university websites received the lowest median score (35%). The information covered within the websites was predominantly poor: 91% (60/66) of the websites received an overall score of ≤74% (mean 53%, SD 18%). The suitability of health information was mostly rated adequate for literacy demand, layout, and learning and motivation of readers. The median readability score for the websites was grade 8.5, which is higher than the government recommendations (<grade 8). Overall, 74% (49/66) of the websites obtained a poor rating for interactivity, measuring active control, 2-way communication, and synchronicity. The most common features found on websites were social media links (61/66, 92%), frequently asked questions (48/66, 73%), and videos (44/66, 67%). Only 14% (9/66) of websites presented culturally responsive information.

**Conclusions:**

Quality, content, readability, and interactivity of websites promoting health behaviors during infancy ranged between poor and adequate. Since the 2015 systematic assessment, there was a slight improvement in the quality of websites but no difference in the Suitability Assessment of Materials rating and readability of information. There is a need for researchers and health care providers to leverage innovative web-based platforms to provide culturally competent evidence-based information based on government guidelines that are accessible to those with limited English proficiency.

## Introduction

### Background

With technological advances and developments, internet access continues to increase [1]. Globally, approximately 4.53 billion people have access to web portals [2,3], with more than half using mobile devices and 38.5% using desktop computers to access the internet worldwide [4]. Increasingly, internet users are making use of the availability of web-based resources, with approximately 4.5% of all internet searches looking for health-related information [5-7]. Recently, nationwide lockdowns, social distancing, and restrictions due to the COVID-19 pandemic have led to an inevitable surge in internet use among individuals, including parents of young children, to seek health information on the web [8,9] and by health care practitioners to assist in service delivery [10,11]. Given that the internet offers considerable opportunities for immediate and easy access to web-based resources, it has become a significant medium for the dissemination of health-related information.

A universal growing demand for, and use of, web-based resources related to child health information is evident [12-15]. The consolidated behavior of using web-based resources for health information is particularly common among new and expecting parents, where they most frequently search the internet for information related to infant nutrition, development, social support, and health symptoms [16-22]. A 2016 Australian survey found that more than 73% of parents with children aged <5 years used websites and web-based forums to access child health–related information [23]. Interestingly, 30% of those parents reported not trusting the information sources [23]. Another recent study in Switzerland showed that 91% of parents with at least one child aged <2 years used the internet to search for information related to their child’s health and development [24]. The most frequently used sources reported were search engines (55%) and websites for parents (47%) [24]. Although the majority used the internet to search for health-related information, a large percentage of parents were skeptical about the trustworthiness of the web-based resources and their ability to correctly interpret the reliability of the health information they found [24]. This highlights the need for, and importance of, accessible websites to present health information accurately while ensuring it can be easily understood by their intended users.

This study updates and expands on a 2015 systematic assessment of infant feeding websites and mobile apps available in Australia [25]. The update of apps has been recently conducted by Cheng et al [26] in 2020. Hence, this study focused on updating and expanding the assessment of websites globally. The 2015 assessment found that 61% of Australian websites were of poor quality, with minimal coverage of infant feeding topics, lack of author credibility, and abstruse readability of content [25]. Since the publication of the 2015 systematic assessment, several other website assessments reviewing information related to infant health behaviors have identified similar findings [27,28]. A Korean study reported that websites were scored poorly when evaluated for availability, quality, and reliability of infant health information on the web [27]. Moreover, a recent analysis of 197 websites addressing preterm infants’ health information also found that the overall quality of websites was low to moderate in terms of reliability and content [28]. Provision of inadequate or incomplete infant health information on the web could result in parental confusion, apprehension, and poorer care for infants when parents are unable to evaluate the accuracy and credibility of the web-based information.

Over the past 10 years, websites have evolved from static *read only* to a more interactive and fully immersive experience [29,30]. Since the 2015 assessment of websites [25], there has been a marked growth in bandwidth levels enabling the design of more sophisticated websites to offer consumer-oriented health information in various interactive ways, such as videos, parent forums, podcasts, and multilingual options that have provided a context to support culturally diverse people across the world [31-38]. In addition, the emergence of artificial intelligence and machine learning since 2016 has given rise to chatbot technology that simulates human-like conversations to provide consumers with support and relevant information [39]. This has been tested and proven to be successful among parents of young infants searching for information related to infant sleep and feeding practices [40].

### Objective

With more parents resorting to web-based sources to seek infant health information, it is imperative for resources on the internet to reflect the latest infant and child health guidelines. Therefore, the aim of this study was to update the 2015 systematic assessment of websites [25] by evaluating the content, suitability, readability, comprehensibility, and quality of information targeting infant nutrition, active play, screen time, and sleep behaviors on websites globally. In addition, this review expanded the 2015 systematic assessment [25] by examining interactivity, features, and cultural considerations of the websites.

## Methods

### Study Design

A systematic search and assessment were conducted to identify and evaluate websites targeting infant feeding, active play, screen time, and sleep behaviors between July 2021 and February 2022. As shown in Table 1, a range of validated tools was used to assess the selected websites. Details of the evaluation tools are described in Multimedia Appendix 1, and the details of the methods are given in Multimedia Appendix 2.

**Table 1:** Comparison of the systematic assessment between the 2015 assessment and this study.

Criteria		Systematic assessment	
		2015	2021
**Website selection**			
	Australian websites only	✓	
	Global websites including Australian websites		✓
**Topic areas**			
	Milk feeding practices (breastfeeding and formula)	✓	✓
	Solid feeding behaviors	✓	✓
	Infant active play		✓
	Infant screen time		✓
	Infant sleep		✓
**Scope, accuracy, and depth of information**			
	Excel spreadsheet built with an assessment criterion of 8 topics and 22 subtopics	✓	
	Comprehensive REDCap^a^ tool built with an assessment criterion of 9 topics and 65 subtopics		✓
**Quality assessment**			
	Quality Component Scoring System	✓	✓
	Health-Related Website Evaluation Form	✓	✓
	Adherence to the Health on the Net code	✓	✓
**Suitability of information**			
	The Suitability Assessment of Material	✓	✓
**Readability**			
	Flesch-Kincaid	✓	✓
	Simple Measure of Gobbledygook	✓	✓
	Consensus based on 7 readability formulas		✓
**Website interactivity and features**			
	The interactivity scale (15 items)		✓
	Interactive features on websites		✓
	Addresses culture		✓

^a^REDCap: Research Electronic Data Capture.

### Stage 1: Website Selection

#### Overview

Websites were identified using the Chrome browser and Google search engine. All cookies and search history were erased from the web browser to ensure no previous web-based activities influenced the search results. The search terms were *Infant feeding, Baby food, Breast feeding, Infant feeding to appetite, Infant formula feeding, Introducing solid foods to baby, Good foods to start baby with no teeth, Best puree for babies, Solids and fussy babies, Solids and milk feeding, Infant active play, Tummy time, Screen time, Infant sleep, Baby co-sleep.* These key terms were identified from questions asked in Facebook groups that consisted of parents with infants and from “related searches” on Google, which was used as a cross-reference to ensure the representativeness of the keywords.

Evidence shows that users concentrate their exploration of websites on the first 10 search results retrieved from a search engine and rarely go beyond the first 2 pages [41]. Hence, the first 30 websites generated from every search term were screened.

#### Inclusion Criteria

We included global websites that used English as a primary language or language option, were free of charge, targeted at parents of infants, and contained information on at least one of the following topics: milk feeding behaviors (breastfeeding, formula feeding, expressing breast milk, feeding to appetite, frequency or timing of feeding, and correct preparation of infant formula, storage of milk, quantity of milk, and transport of milk), solid food feeding behaviors (age of introduction, types of food introduced, and food allergies), infant activity (“tummy time,” infant play, and movement), and infant screen time or infant sleep (bedtime routine, recommended hours of sleep, and cosleep) regardless of whether the websites addressed other content or age groups.

#### Exclusion Criteria

We excluded websites that had no information on one of the topics of interest listed in the inclusion criteria; were newspapers; were electronic books; required a password; had a payment fee; or had a link that redirected user to a scientific article, podcast, or downloadable Microsoft Word document and PDF document.

The first author screened all the websites for eligibility using predefined inclusion and exclusion criteria. Any uncertainties or disagreements regarding the inclusion of websites in the study were cross-checked by researchers ST, LMW, LB, and CR in a group meeting and discussed until consensus was reached.

### Stage 2: Website Evaluation

#### Scope, Accuracy, and Depth of Information

Scope, accuracy, and depth of information were evaluated using a newly built tool on a password-protected database (REDCap [Research Electronic Data Capture]; Vanderbilt University) that was based on the Australian government’s guidelines on infant feeding [42], physical activity [43], and sleep [44]. The tool consists of 11 broad topics with 65 subtopics on encouraging and supporting breastfeeding; initiating, establishing, and maintaining breastfeeding; management of common breastfeeding problems; expressing and storing breast milk; breastfeeding in specific situations; infant formula; solid food introduction; encouraging infant active play; screen time; and infant sleep behaviors. Each subtopic was scored as correct (+1), incorrect (−1), not addressed (0), or not applicable (which was not counted in the denominator of the overall score). For subtopics that were partially addressed, a partially complete (+0.5) score was given. A summary section score was automatically calculated for each topic, and a final overall score was generated after the assessment of all the content for the 11 topic areas. Overall scores were summarized as excellent (≥90%), adequate (75%-89%), or poor (≤74%) using the criteria from the Health-Related Website Evaluation Form (HRWEF) [45] similar to the updated assessment of apps [26].

#### Website Quality

Website quality was evaluated using the same validated tools as the 2015 assessment: the Quality Component Scoring System (QCSS) [46,47], the HRWEF [45], and the adherence to the Health on the Net Foundation Code of Conduct (HONcode) [48]. The QCSS is an instrument designed to offer scores on ownership, authorship, author qualification, purpose, attribution (references provided for requiring statements), interactivity, and currency of posting and revision. The sum of scores generates a final score summarized as excellent (80%-100%), very good (70%-79%), good (60%-69%), fair (50%-59%), or (poor 0%-50%). The HRWEF tool can be used by health professionals and patients to assess the appropriateness of websites. It consists of 30 items where each criterion is rated on a 3-point scale, scored as not applicable (score=0), disagree (score=1), or agree (score=2). It is divided into 7 main sections assessing the content, accuracy, author, currency, audience, navigation, and external links. An overall score was designated as excellent (90%-100%), adequate (75%-89%), or poor (0%-75%). Moreover, the HONcode certification validates and certifies the quality of the medical information provided on the internet.

#### Suitability of Information

The Suitability Assessment of Materials (SAM) tool [49] was used to assess the appropriateness of health information materials by considering characteristics such as content, graphics, literacy level, layout, typography, and cultural appropriateness of the websites. Each of the 22 items was rated as superior (rating +2), adequate (rating +1), not suitable (rating 0), or not applicable. Scores were summed to yield an overall percentage for the website reported as superior (70%-100%), adequate (40%-69%), or not suitable (0%-39%).

#### Readability

Readability tools were used to assess the difficulty of reading the written texts on the websites. The Flesh Kincaid test (F-K) [50], Simple Measure of Gobbledygook (SMOG) [51], and readability consensus based on 7 readability formulas (Flesch Reading Ease score, Gunning Fog, F-K, SMOG, the Coleman-Liau Index, Automated Readability Index, and Linsear Write Formula) [52] were used. The reviewers assessed the readability by selecting multiple written sections from each website and inserting it into a web-based readability calculator [52] that calculated F-K, SMOG, and readability consensus scores. In addition, as an item of the SAM tool, readability was also assessed using SMOG and rated as superior (grade 5 or lower), adequate (6th-8th grade level), or not suitable (grade 9 or higher). The Australian government recommends aiming for a lower than grade 8 reading level for health information [53-55], whereas the American Medical Association recommends education materials to be written at grade 6 reading level or lower [56].

#### Website Interactivity and Features

A validated interactivity scale was used as an individual consumers’ perceptual assessment of websites in a previous study, which asked undergraduate business students ranging from age 19 to 40 years were asked to browse and rate websites using 15 items based on their personal experience [57]. The 15 items measuring active control (control over what users can do and see on the websites), two-way communication (ease of communication and offering feedback on the website), and synchronicity (website responsiveness to input and obtaining instantaneous information) were adopted for the purpose of this study.

A 3-point Likert scale was created to score each item as follows: agree (score=2), partially agree (score=1), or disagree (score=0), and an average score for all components was calculated. Interactivity scale was summarized as excellent (≥90%), adequate (70%-89%), or poor (≤69%).

Interactive aspects and features were also assessed by looking at whether the website was functional on a smartphone screen, had an associated app, addressed ethnicity, and included language options, paid features, search functions, games, videos, podcasts, chatbot, question and answer forum, quizzes, animation, a feedback form, slide shows, ratings, frequently asked questions section, recipes, read out loud options, navigation menu, social media links, acceptable page speed, webinars, or other.

### Statistical Analysis

#### Interrater Reliability

Authors DJ and HC undertook interrater reliability (IRR) checking. A random 10% sample of all websites (n=7) were selected—the coding by DJ and HC were compared, and an IRR score was generated. Discrepancies were discussed until reviewers reached a consensus on their final ratings. Any disagreements were resolved by a third reviewer (ST).

IRR was calculated for the readability scores, quality of content scores, SAM, and the evaluation of information content using intraclass correlation coefficients (ICCs), with a high ICC value (maximum 1.0 indicating no variance in the scoring between different assessors, whereas ≥0.5 was moderate, ≥0.70 was good, and ≥0.80 indicated excellent reliability).

#### Software Used

Data were transferred from REDCap to SPSS for MacBook (version 27.0; IBM Corp), where statistical analyses were performed. The ICC values calculated for content, HRWEF, QCSS, interactivity, and SAM were 0.5, 0.6, 0.6, 0.7, and 0.7, respectively, indicating a moderate to good level of consistency for the rating measurements. As the readability grades were calculated using computerized software, interrater consistency was not measured. The reviews discussed discrepancies by re-evaluating the websites together to ensure scoring consensus.

## Results

### Screening Process

**Figure 1:**
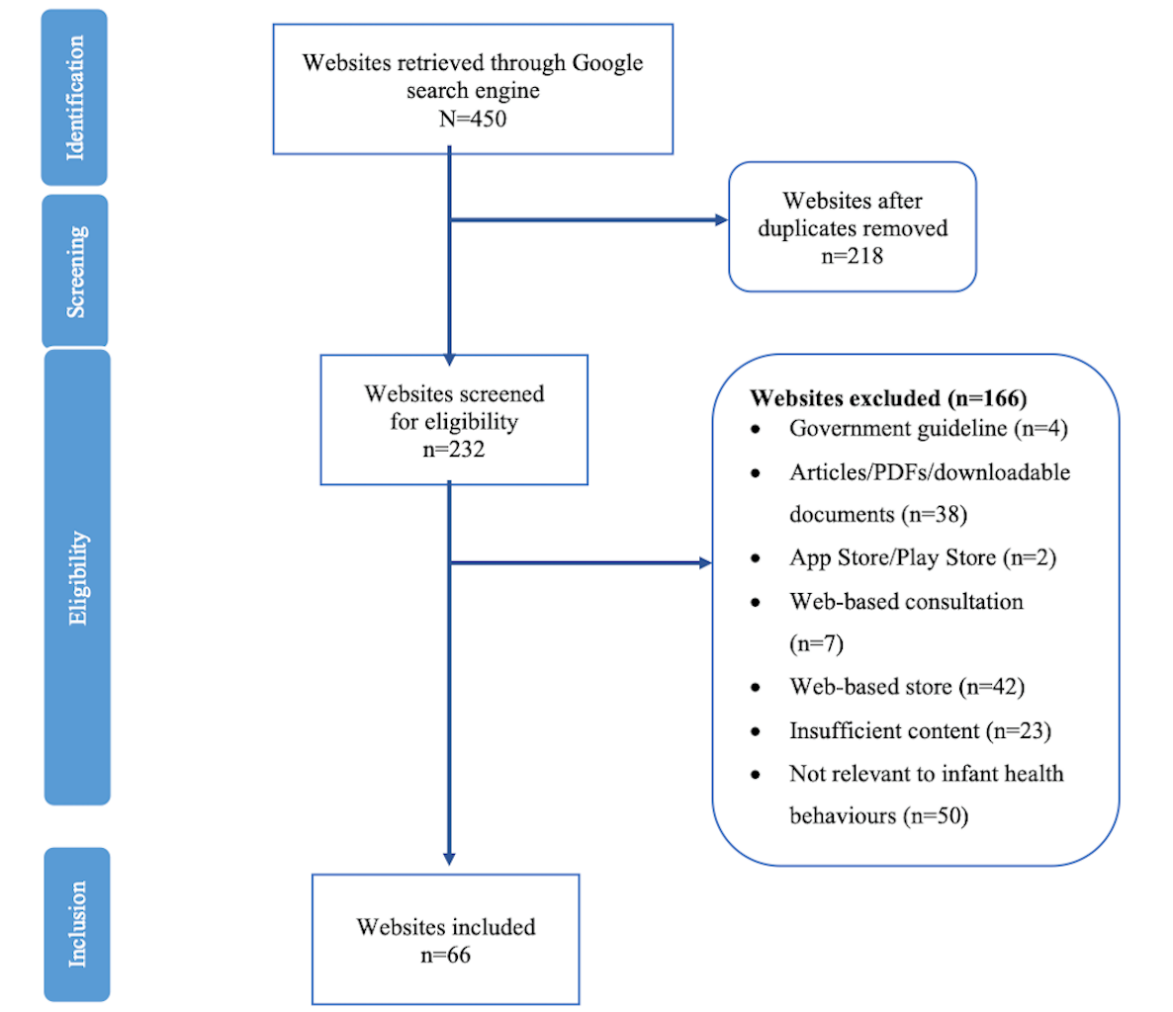
Diagram of website selection process.

As shown in Figure 1, a total of 450 global websites were reviewed between August 2021 and February 2022. The removal of 218 duplicate websites left a total of 232 unique websites. Of these, 66 websites met the inclusion criteria and were eligible to be evaluated. The remaining 166 websites were excluded, as 50 were not relevant to infant health behaviors; 38 were articles, PDF documents, or downloadable documents; 42 were a web-based shops; 23 had insufficient content; 4 were government guidelines; 7 offered web-based consultations, and 2 were infant-related apps on Google Play and App Store.

### Scope, Accuracy, and Depth of Information

#### Scope and Depth of Subtopics

The scope and depth of the information covered in the subtopics were predominantly poor, with 91% (60/66) of websites obtaining an overall score of ≤74%, whereas only 9% (6/66) of websites were rated as adequate.

The overall mean rating of all websites was poor (53%, SD 18%; IQR 40%-67%; Table 2). Expressing, feeding, and storage of expressed breast milk, preparing and feeding infant formula practices, and monitoring infant’s progress topics had the lowest mean scores of 33%, 43%, and 49%, respectively. For information on infant sleep recommendations and bedtime practices, active play, screen time, and breastfeeding recommendations, correct advice was mostly reported as reflected by their mean scores of 73%, 70%, and 66%, respectively. Only 17% (10/58) of websites fully addressed infant feeding to appetite by encouraging responsiveness to infant hunger and satiety cues, not pressuring the baby to finish the bottle, feeding to appetite or baby-led feeding, avoiding bottle propping and bottle use in bed, and benefits of allowing infants to self-regulate their own appetite. Infant feeding to appetite was partially addressed by 59% (34/58) of the websites by highlighting a few of the above-mentioned points (Table 2 and Multimedia Appendix 3).

**Table 2:** The quantitative scope and depth of information based on Australian infant feeding and physical activity guidelines on all websites (N=66).

Topics addressed and websites^a^		Values, mean (SD)	Values, median
**Breastfeeding**			
	Breastfeeding recommendations (n=58)	66 (21)	71
	Physiology of breast milk and breastfeeding (n=55)	62 (21)	62.5
	Monitoring infant’s progress (n=56)	49 (26)	50
**Breastfeeding, common problems, and their management**			
	Maternal factors affecting breastfeeding (n=54)	52 (26)	54
	Infant factors affecting breastfeeding (n=54)	50 (26)	50
**Expressing and storing breast milk**			
	Expressing, feeding, and storage of expressed breast milk (n=50)	33 (32)	30
**Infant formula**			
	Preparing and feeding infant formula practices (n=58)	43 (19)	46
**Introducing solids**			
	Solid introduction and foods and beverages not suitable for infants (n=58)	50 (23)	50
**Infant activity**			
	Active play and screen time (n=52)	70 (30)	77.5
**Infant sleep**			
	Cosleep recommendations (n=53)	61 (21)	62.5
	Sleep recommendations and bedtime practices (n=54)	73 (29)	75
**Overall content**			
	Overall scope and depth of information (n=66)	53 (18)	55

^a^Not all websites included information on all subtopics.

#### Subtopics Addressed

Subtopics that were most frequently correctly addressed on websites were recommendation to exclusively breastfeed till 6 months of age and continue breastfeeding with appropriate complementary food till 12 months of age and beyond (45/58, 77% of websites); natural patterns of breastfeeding 8 to 12 times over 24 hours (42/55, 76% of websites); postnatal breastfeeding advice to seek support from lactation consultants, midwives, or doctors (40/58, 68% of websites); tummy time recommendations (40/52, 76% of websites); and cosleeping in a separate cot but the same room as parents for the first 6- to 12-month recommendation (39/53, 73% of websites).

#### Subtopics Not Addressed

Subtopics that were most often not addressed on websites were supplemental requirements for infants on a vegan diet (30/58, 51% of websites); sterilization and proper use of hand pumps (30/50, 60% of websites); factors affecting initiation of lactation after birth (30/54, 55% of websites); importance and awareness of baby-friendly hospital initiative (27/58, 46% of websites); and correct water temperature for preparing infant formula and risk of infection from *Cronobacter sakazakii* bacteria (26/58, 44% of websites).

#### Subtopics Incorrectly Addressed

Subtopics that were most frequently incorrectly addressed were storage of freshly expressed, thawed, or used breast milk (22/50, 44% of websites); correct selection of infants’ first foods (6/58, 10% of websites); and correct preparation of infant formula (5/58, 8% of websites).

### Assessment of Website Quality

#### Using HRWEF

A majority of the websites attained an adequate rating (49/66, 74%) for the quality of the websites using the HRWEF tool. Although 20% (13/66) of the websites were rated as excellent, the remaining 3% (4/66) of websites received a poor scoring, 3 of which were commercial.

The overall HRWEF mean percentage score was 85% (SD 5.98%). The questions with the highest scores addressed the organization of the site, navigation, internal link, and the type of audience the author targeted. Conversely, the questions with the lowest scores were related to dates of publication and revision of content (Multimedia Appendix 3).

#### Using the QCSS

From the quality evaluation conducted using the QCSS tool, 8% (5/66) were rated as excellent, 21% (14/66) as very good, 32% (21/66) as good, 11% (7/66) as fair, and 29% (19/66) were rated as poor. The overall mean QCSS score was 60 (SD 18; Multimedia Appendix 3).

In comparison with websites that scored excellent, poorly rated websites failed to provide references, author qualifications, and currency of content. A total of 32 websites stated that the author was a health care professional, whereas 30 websites clearly listed the name of the person supplying the information and author qualification. In addition, 31 websites had failed to display references for requiring statements. Only 3 websites presented the dates of original posting and revision.

A total of 9 websites stated they had acquired the HONcode certification demonstrating the intent of offering quality health information to meet ethical standards.

#### Quality (QCSS) by Organization

**Figure 2:**
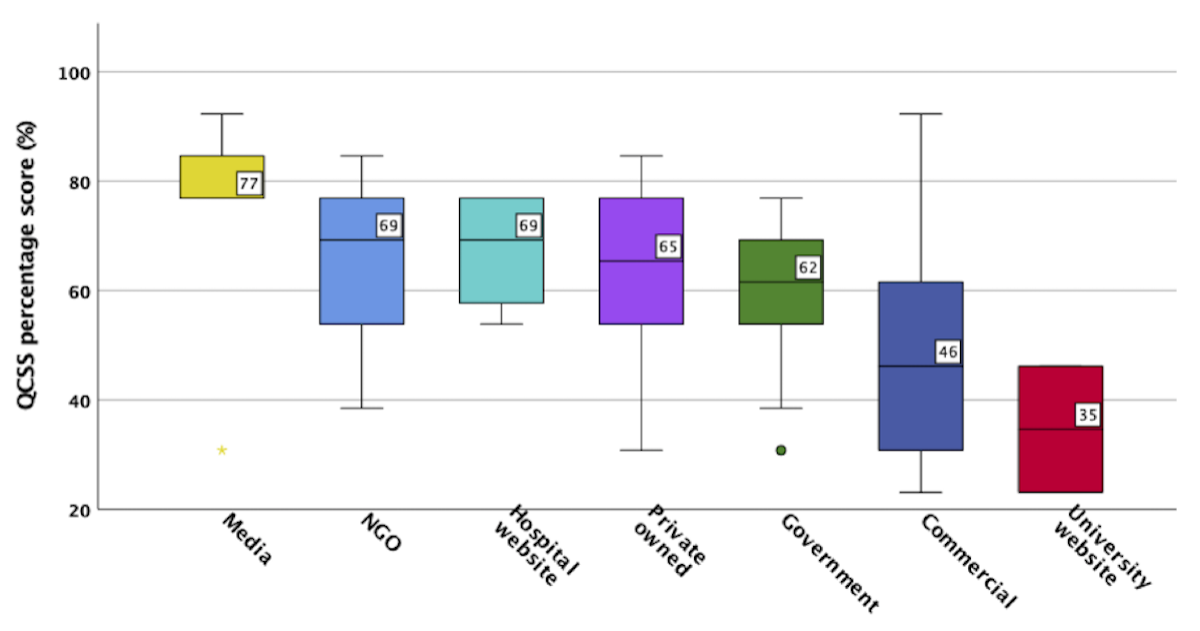
Quality Component Scoring System (QCSS) by type of organization. NGO: nongovernmental organizations.

Figure 2 shows the quality of the websites by the type of organization as measured by QCSS. This ranged from poor to very good. Media-related sources received the highest median score of 77% (very good), followed by nongovernmental organizations, hospital websites, and privately owned and government websites, which received a score corresponding to good. Commercial websites had a mean score of 46% (fair), and university websites received the lowest median score of 35% (poor).

### Assessment of Suitability of Website Information by SAM

Overall, 3% (2/66 assessments) of websites were rated *superior* for suitability of health information, 82% (54/66 assessments) of websites as *adequate*, and 15% (10/66 assessments) of websites *not suitable,* as shown in Table 3 (Multimedia Appendix 3).

Very few websites were rated superior on literacy demand, such as writing style, context, and vocabulary used. Overall, 15% of websites provided culturally appropriate visual aids based on the consumers they were targeting. There were variations in the type of images used in the resources. Some images depicted different sex, race, color, religion, and age, whereas others targeted specific cultural groups such as Indian and Aboriginal and Torres Strait Islander. Only one of the sites addressed the cultural specificity of information relating to experience, language, or provision of examples to patients from diverse sociodemographic backgrounds.

Overall, 21% (14/66) of websites had the option to be translated into a language other than English, such as Arabic, Spanish, Hindi, or Bengali. Only 14% (9/66) presented information that addressed culture in texts or images. The culturally appropriate information varied between fasting tips and breastfeeding in Islam, Christianity or Judaism, fasting and pregnancy tips, multiracial graphics, and recipes.

Many websites provided a clear layout of information and easily understandable cover graphics that clearly portrayed the purpose of the material. Most topics were subdivided to improve readers’ self-efficacy, which was rated as *adequate*; for instance, infant sleep was subdivided into quiet playtime, bedtime routine, safety sleep practices, and infant sleep recommendations.

**Table 3:** Website scores based on the Suitability Assessment of Materials (SAM) criteria.

			SAM scores (evaluations), n (%)						
			Not suitable		Adequate		Superior		Not applicable
**Content**									
	Purpose is evident	1 (1)		30 (45)		35 (53)		—^a^	
	Content about behaviors	2 (3)		59 (89)		5 (8)		—	
	Limited to essential information	4 (6)		40 (61)		22 (33)		—	
	Summary and review	46 (70)		16 (24)		3 (5)		1 (1)	
**Literacy demand**									
	Reading grade level	31 (47)		31 (47)		3 (5)		1 (1)	
	Writing style with active voice	5 (8)		55 (83)		6 (9)		—	
	Vocabulary uses common words	5 (8)		56 (84)		5 (8)		—	
	Context given first	11 (17)		47 (71)		8 (12)		—	
	Headers or topic captions	1 (1)		21 (32)		44 (67)		—	
**Graphics**									
	Purposeful cover graphic	9 (14)		26 (39)		30 (46)		1 (1)	
	Appropriate type of illustrations	9 (14)		22 (33)		6 (9)		29 (44)	
	Relevance of illustrations	11 (17)		19 (29)		8 (12)		28 (42)	
	Lists, tables, graphs, and charts explained	7 (11)		45 (68)		5 (8)		9 (14)	
	Captions used for graphics	9 (14)		41 (62)		11 (17)		5 (8)	
**Layout and typography**									
	Layout factors	6 (10)		58 (88)		2 (3)		—	
	Typography	2 (3)		59 (89)		5 (8)		—	
	Subheadings used	0 (0)		42 (64)		24 (36)		—	
**Learning, stimulation, and motivation**									
	Interaction with readers used	22 (33)		44 (67)		0 (0)		—	
	Modeled and specific behaviors	16 (24)		45 (68)		5 (8)		—	
	Self-efficacious tasks and behaviors	5 (8)		57 (86)		4 (6)		—	
**Cultural appropriateness**									
	Cultural match	0 (0)		1 (1)		0 (0)		65 (99)	
	Cultural image and examples	0 (0)		8 (12)		2 (3)		56 (85)	

^a^Not included in the overall score.

### Assessment of Website Readability

Very few websites met the Australian Federal government’s recommended level for written health information of lower than a grade 8 reading level: 29% (19/66) of websites (SMOG), 20% (13/66) of websites (F-K web-based tool), and 12% (8/66) of websites (consensus tool). In 2 of the websites assessed for interreliability, the readability scores ranged from 8 to 11 and 7 to 14 between the researchers depending on the varying content selected for assessment (Multimedia Appendix 4).

The median readability grades were 8.5 (IQR 7-10), 9 (IQR 8-11), and 10 (IQR 8-11) using the SMOG formula, web-based F-K calculator, and consensus calculator, respectively. There was a good correlation among reading grade scores across the readability measures (*P*<.001; 2-tailed).

### Assessment of Website Interactivity and Features

Table 4 presents the results of the websites interactivity scores in terms of active control, two-way communication, and synchronicity.

**Table 4:** Interactivity scores of websites (N=66).

		Agree, n (%)
**Active control**		
	I felt that I had a lot of control over my visiting experiences at this website	17 (25)
	While I was on the website, I could choose freely what I wanted to see	23 (34)
	While surfing the website, I had full control over what I can do on the site	21 (31)
	While surfing the website, my actions decided the kind of experiences I got	24 (36)
**Two-way communication**		
	The website is effective in gathering visitors’ feedback	6 (9)
	This website facilitates two-way communication between the visitors and the site	7 (10)
	It is easy to offer feedback to the website	13 (19)
	The website makes me feel it wants to listen to its visitors	9 (13)
	The website encourages visitors to talk back	8 (12)
	The website gives visitors the opportunity to talk back	18 (27)
**Synchronicity**		
	The website processed my input very quickly	16 (24)
	Getting information from the website is very fast	15 (22)
	I was able to obtain the information I want without any delay	10 (15)
	When I clicked on the links, I felt I was getting instantaneous information	26 (39)
	The website was very fast in responding to my requests	18 (27)

The overall interactivity of websites was predominantly poor (49/66, 74%). The remaining 26% (17/66) of websites were marginally adequate with no websites obtaining an excellent rating. More than half the websites acquired an incomplete score for active control resulting from slow loading web pages and the inability of site search engines to return relevant results effectively. Very few websites encouraged visitors to talk back or facilitated 2-way communication between the visitors and the site. As for synchronicity of the sites, approximately one-fourth of websites received a full score (agree) for their ability to process input and respond to requests promptly (Multimedia Appendix 4).

The most common features found on websites were social media links (61/66, 92%), frequently asked questions (48/66, 73%), videos (44/66, 67%), and recipes (35/66, 53%), whereas language options, webinars, question and answer forums, chatbots, read out loud function, slide shows, animation, and games were less common. Moreover, 80% (53/66) of websites had additional features such as text font size options, tools (eg, ovulation calculator, pregnancy calculator, and parenting tools), download and print page content option, and YouTube accounts. Overall, 47% (31/66) of websites had associated apps on Google Play and Apple Store. Log-in options for personalized health information were presented on 40% (26/66) of the websites.

## Discussion

### Principal Findings

In this review, we systematically assessed 66 websites that reported health information related to infant nutrition, active play, screen time, or sleep behaviors. This review extends on the existing 2015 assessment by providing 2 main conceptual contributions. First, it covers the quality, content, suitability, readability, and comprehensibility of web-based infant health information at a global level. Second, it assesses the interactivity, features, and cultural considerations of webpages. In this section, we discuss the principal findings, comparison to prior work, implications for future practice, and then outline the strengths and limitations of this review.

This study found that the information content of the websites was overall poor in terms of scope and depth of information, which was similar to the findings of the previous 2015 assessment. Approximately one-third of the websites reported different advice on storage of expressed breast milk; for instance, “freshly expressed breast milk can be stored safely in the refrigerator for up to five days.” This information was contrary to the Australian guidelines on infant feeding that stated storage of expressed breast milk should not exceed 72 hours in the fridge [42]. This was due to the development of some websites in other countries, such as America or Europe, where guidelines differ from that of Australia [58,59].

The quality of websites was generally adequate when evaluated by HRWEF and mostly ranged from poor to good when rated by the QCSS tool. These findings are consistent with those from previous studies [60-63], which evaluated a range of health information available on the web using similar tools. This study also highlights that the quality of websites in terms of ownership, authorship, author qualification, purpose, referencing statements, and currency of information was the highest among media, nongovernmental organizations, and hospital websites and the lowest among university websites. This is an important finding, given that parents view university sites as a high-quality, reliable, and credible source of information [64]. It is vital that the health information on websites is continuously updated to meet the latest guidelines with relevant currency, authorship, qualification, and supporting attribution statements. This in turn will provide readers with the clarity they need to assess the quality of web-based health information and identify reputable websites. Moreover, websites with outdated information and no supporting statements can mislead readers, resulting in adverse health consequences [65-67].

Using the SAM tool, we found that website information on infant health behaviors was generally adequate. This finding is consistent with that of other studies on the SAM [68-70]. Despite the overall adequate suitability ratings of information on the selected websites, there were marked limitations in terms of cultural appropriateness, literacy demand, and illustrations. This highlights the issue that web-based infant health information rarely considered the needs of people from non–English-speaking ethnic groups and how they may interpret or apply the health information. This is unfortunate, given the ubiquitous nature of the internet. Given the increase in cultural diversity within Australia and abroad, it is important to consider cultural appropriateness of information and provide culturally and ethnically diverse consumers the capacity to access, understand, and use health information to make well-informed health decisions [71-73].

In regard to readability, this study highlighted that most websites were at readability levels beyond the ideal level of lower than grade 8. This finding was also reflected in previous studies [74-76]. Notably, a difference of 4 to 6 grades was observed between the interreliability readability grades of 2 websites scored by the 2 researchers DJ and HC. This reflected the inconsistent readability levels across various webpages within a website. Readability and health literacy play an integral part in information accessibility and usability [77]. The readability formulas are based on the number of words, sentence length, and number of syllables per word. Therefore, using simpler words, shorter sentences, pictures, videos, and co-design methods as per the Australian Commission for Safety and Quality in health care are important considerations for writing health information for consumers [78].

Furthermore, readability levels of web-based health information should be tested for consistency and presented in an easy-to-read format providing access to people with low health literacy.

The overall interactivity of website functions was poor. In addition, interactive features were mostly common among media and commercial websites and least common among government websites. According to the World Health Organization, various provision methods of providing health information are important to increase accessibility and achieve positive health outcomes [79]. Multiple patient-focused interventions have reported that various health information formats, such as videos, audios, and infographics, have contributed to the improvement in parental knowledge, satisfaction, and health outcomes [80-82]. Hence, it is crucial for credible websites such as government owned to make the wealth of information available on the web interactive and accessible to increase consumer engagement and use of reliable sources.

### Comparison With Prior Work

In comparison with the 2015 review, 55 new websites were assessed in this review, whereas 11 were common across both studies. It is important to note that the first 30 websites generated from every search term used resulted in a range of global websites for consumers to access. Hence, it is vital for education to be provided to parents of young children and health professionals that will enable them to determine the quality and credibility of web-based health information as accuracy is critical, especially in the first 1000 days of life [83,84].

Furthermore, several websites from the 2015 review were excluded for reasons such as their web-based content had been removed or they no longer exist. A potential reason may be due to the cost and maintenance of websites that were developed with limited funding. A systematic review of factors that influence eHealth reported that ongoing maintenance costs were barriers for several studies [85]. Another reason may be due to the evolution of websites over time and the inability of static websites to enable and host new features [30].

To replicate and compare results from the 2015 assessment, the same validated tools were used to assess the quality, suitability, and readability of infant health websites. Interestingly, the results of the new eligible websites did not greatly differ from the results of the 2015 systematic assessment [25]. There was a slight improvement in the quality of websites from poor to adequate or good measured by HRWEF and QCSS since 2015. However, the suitability rating of websites in both assessments measured by SAM was the same. Similarly, the readability of written health information in the majority of the websites did not meet government recommendation in both assessments as well. Although websites have evolved since 2015, minimal to no improvements have been identified in this review. One reason could be due to the lack of user involvement in the website design. A recent systematic review reported that a considerable number of studies raised concerns that involving users in technology design can be fairly demanding and requires time and effort [86]. Another reason may be the lack of use of validated tools such as the ones used in this study to ensure optimized quality during the development of websites.

Identifying cultural considerations, interactivity, and features of web pages is a value added to this study. We found that very few websites addressed culture or had interactive features such as multilingual options, chatbots, or read out loud functions. With the rise in immigrants from culturally and linguistically diverse communities [87], more and more people are facing access barriers to health information and eHealth services due to the lack of language support and culturally appropriate health information through the internet [88-90]. This demonstrates the need to engage end users throughout the web development to ensure high-quality outcomes and meet consumers’ needs and expectations.

### Implications for Practice

With the wide spread of internet use and wealth of information available on the web [91], it is imperative for parents and health professionals to be guided to the optimal and most accurate sources of information. Websites should be screened for authorship, ownership, information date, and HONcode certification before use. There is a need for web developers to bear in mind their end users. One way to overcome this challenge is by involving consumers in website development through co-design workshops [92]. This will ensure developers get a good understanding of end users’ access requirements, literacy demands, preference for alternative presentation formats of information, and cultural considerations. An establishment of a regulatory body is also recommended to ensure that newly developed websites are built on validated tools and that all websites comply with government guidelines standards.

### Strengths and Limitations

To the best of our knowledge, this is the first study to evaluate website interactivity, features, and cultural considerations for web-based infant health information. This review provides a comprehensive global overview of the available web-based information about infant health behaviors and identifies ways for improvement.

Although this study adhered to a rigorous systematic search process, there were several limitations. First, most websites included in this evaluation differed from that which was evaluated in the 2015 review. Hence, this demonstrates the dynamic nature and constant change of the internet. Thus, the website search during this review reflected a period that could potentially change. In addition, the website search was conducted in the English language using Google. Although Google is a highly used search engine, we acknowledge that some international users have access restraints [93]. Therefore, the results may not have identified websites present on other search engines or written in other languages. Furthermore, deleting cookies and search histories was intended to reduce unknown bias in the search strategy. However, it is acknowledged that the likelihood of most web users doing this is unlikely, and their searches might identify sites of which we were unaware.

Another limitation is that the assessment criteria used Australian guidelines. Therefore, there was a potential for websites following non-Australian guidelines to obtain an incorrect score on a few subtopics. Moreover, the interactivity scale used was originally meant to capture consumers’ perceptual assessment of websites. However, due to the lack of published validated tools used to measure interactivity of websites, the interactivity scale was adopted for the purpose of this study. Thus, the interactivity scale used was a subjective measurement based on the researcher’s experience on the websites. Nevertheless, the tools used offer a standardized way to best capture the quality and interactivity of web-based information.

### Conclusions

As more parents seek web-based guidance on infant health behaviors globally, there remains a significant concern on the quality, readability, interactivity, and accessibility of websites promoting health behaviors during infancy. This systematic assessment revealed that there is a need for researchers and health care providers to leverage innovative web-based platforms to provide culturally responsive evidence-based information accessible to those with limited English proficiency. Furthermore, a focus is needed on continuously updating existing health websites in addition to recommending an establishment of a regulatory body to ensure compliance with government standards. Moreover, the development of new eHealth technology should be based on validated tools to ensure the optimal quality of websites.

## Appendix

Multimedia Appendix 1 Website evaluation tools.

Multimedia Appendix 2 Protocol.

Multimedia Appendix 3 Summary score for all websites.

Multimedia Appendix 4 Supplementary tables and graphs.

## Abbreviations

F-K: Flesch-Kincaid test
HONcode: Health on the Net Foundation Code of Conduct
HRWEF: Health-Related Website Evaluation Form
ICC: intraclass correlation coefficient
IRR: interrater reliability
QCSS: Quality Component Scoring System
REDCap: Research Electronic Data Capture
SAM: Suitability Assessment of Materials
SMOG: Simple Measure of Gobbledygook
