# Supplementary material for: Interactivity, Quality, and Content of Websites Promoting Health Behaviors During Infancy: 6-Year Update of the Systematic Assessment
Source: J Med Internet Res. 2022 Oct 7;24(10):e38641. doi: 10.2196/38641 (PMC9587494; doi:10.2196/38641)
Supplement: Multimedia Appendix 1 [file jmir_v24i10e38641_app1.docx]

# Appendix 1

## Health-Related Web Site Evaluation Form

**I. Web site information**

Title of site:_____________________________________________________________________________

Subject of site:_____________________________________________________________________________________________

Web site address:___________________________________________________________________________________________

Whom do you think is the intended audience?_____________________________________________________________________

What do you think the objective is for this site?_________________________________________________________________________________________________________________________________________________________________________________________________________________________________________

Circle the number which you feel best represents the site: 1 = disagree, 2 = agree, 0 = not applicable (N/A). Add up the total points scored for each page at the bottom of each page.

**II. Content**

|  | **Disagree**  **1** | **Agree**  **2** | **N/A**  **0** |
| --- | --- | --- | --- |
| 1. The purpose of the site is clearly stated or may be clearly inferred. |  |  |  |
| 1. The information covered does not appear to be an “infomercial” (i.e., an advertisement disguised as health education.) |  |  |  |
| 1. There is no bias evident. |  |  |  |
| 1. If the site is opinionated, the author discusses all sides of the issue, giving each due respect. |  |  |  |
| 1. All aspects of the subject are covered adequately |  |  |  |
| 1. External Links are provided to fully cover the subject |  |  |  |

**III.Accuracy**

|  | **Disagree**  **1** | **Agree**  **2** | **N/A**  **0** |
| --- | --- | --- | --- |
| 1. The information is accurate (if not sure, circle 0). |  |  |  |
| 1. Sources are clearly documented |  |  |  |
| 1. The web site states that it subscribes to HON code principles   Page score_________ |  |  |  |

**IV.Author**

|  | **Disagree**  **1** | **Agree**  **2** | **N/A**  **0** |
| --- | --- | --- | --- |
| 1. The site is sponsored by or is associated with an institution or organization. |  |  |  |
| 1. For sites created by an individual, author’s/editor’s credentials (educational background, professional affiliations, certifications, past writings, experience) are clearly stated. |  |  |  |
| 1. Contact information (email, address, and/or phone number) for the author/editor or webmaster is included. |  |  |  |

**V. Currency**

|  | **Disagree**  **1** | **Agree**  **2** | **N/A**  **0** |
| --- | --- | --- | --- |
| 1. The date of publication is clearly posted. |  |  |  |
| 1. The revision date is recent enough to account for changes in the field. |  |  |  |

**VI.Audience**

|  | **Disagree**  **1** | **Agree**  **2** | **N/A**  **0** |
| --- | --- | --- | --- |
| 1. The type of audience the author is addressing is evident (academic, youth, minority, general, etc.). |  |  |  |
| 1. The level of detail is appropriate for the audience. |  |  |  |
| 1. The reading level is appropriate for the audience |  |  |  |
| 1. Technical terms are appropriate for the audience. |  |  |  |

**VII. Navigation**

|  | **Disagree**  **1** | **Agree**  **2** | **N/A**  **0** |
| --- | --- | --- | --- |
| 19. Internal links add to the usefulness of the site. |  |  |  |
| 20. Information can be retrieved in a timely manner. |  |  |  |
| 21. A search mechanism is necessary to make this site useful. |  |  |  |
| 22. A search mechanism is provided. |  |  |  |
| 23. The site is organized in a logical manner, facilitating the location of information.  24. Any software necessary to use the page has links to download software from the Internet. |  |  |  |

**VIII.External Links**

| 25. Links are relevant and appropriate for this site. |  |  |  |
| --- | --- | --- | --- |
| 26. Links are operable. |  |  |  |
| 27. Links are current enough to account for changes in the field. |  |  |  |
| 28. Links are appropriate for the audience (e.g. site for the general public do not include links to highly technical sites). |  |  |  |
| 29. Links connect to reliable information from reliable sources |  |  |  |
| 30. Links are provided to organizations that should be represented |  |  |  |

Page Score ________

**Total score________**

Total number of possible points ________

Percentage of total points ________

Total the number of points possible (the number of questions scored with either disagree or disagree multiplied by two). Divide your total score by the total number of points possible to determine the overall rating of this web site.

*At least 90% **Excellent**: This web site is an excellent source of patient of total information. Patients will be able to easily access and understand

possible the information contained in this site. Do not hesitate to points. Recommend this site to your clientele.

*At least 75% **Adequate**: While this web site provides relevant information and of total can be navigated without much trouble, it might not be the best site possible available. If another source cannot be located, this site will provide points good information to your patient. Care should be taken to discuss with your patient what information was found on this web site and what information is still needed.

*< 75% of **Poor**: This site should not be recommended to your patients total Validity and reliability of the information cannot be confirmed. possible All information on the site might not be accessible. Look for points another web site to prevent false or partial information from being read

## Quality Component Scoring System

| **Quality Component Scoring System** | |
| --- | --- |
| **Criteria** | **Score** |
| **Ownership** |  |
| No indication of ownership/sponsorship | 0 |
| Ownership/sponsorship clearly stated | 1 |
| **Purpose** |  |
| No statement of purpose | 0 |
| Purpose stated as educational but the financial profit from use of the site exists | 1 |
| Distinction is made as to whether the information provided is for commercial purposes or educational purposes, or both | 2 |
| **Authorship** |  |
| No indication of authorship | 0 |
| All other indications of authorship | 1 |
| Name of person(s) supplying information clearly provided | 2 |
| **Author Qualification** |  |
| Author has no officially recognised experience in the field or no such information is provided | 0 |
| Information about the author’s professional experience is vague, or if the author has no professional experience but has direct personal experience. | 1 |
| If author is a healthcare professional | 2 |
| **Attribution** |  |
| No references provided for requiring statements | 0 |
| References are provided for same, but not all, statements requiring factual information | 1 |
| Attribution for all statements conveying factual information is present | 2 |
| **Interactivity** |  |
| No contact provided | 0 |
| Telephone number, email, or mailing address provided | 1 |
| Clear invitation to comment or ask questions by an email address or link to a form | 2 |
| **Currency** |  |
| No date provided | 0 |
| Date of original posting provided, but no information about the date of last revisal or frequency of updates | 1 |
| Date of original posting and date of last revisal or frequency of updates clearly stated | 2 |

## Suitability Assessment of Material (SAM)

The scoring system used for each item measured included not suitable (0), adequate (1), or superior (2), and each website and app was given a final rating of superior (70-100%), adequate (40-69%), or not suitable (0-39%).

| **Evaluation Criteria** | **Superior x2** | **Adequate x1** | **Not Suitable x0** | **Not relevant to source** |
| --- | --- | --- | --- | --- |
| 1. **Content** |  |  |  |  |
| Purpose is evident |  |  |  |  |
| Content about behaviour information |  |  |  |  |
| Scope is limited |  |  |  |  |
| Summary of website is available |  |  |  |  |
| 1. **Literacy demand** |  |  |  |  |
| Reading grade level |  |  |  |  |
| Writing style, active voice |  |  |  |  |
| Vocabulary uses common words |  |  |  |  |
| Context is given first |  |  |  |  |
| Learning aids via “road signs” |  |  |  |  |
| 1. **Graphics** |  |  |  |  |
| Cover graphic shows purpose |  |  |  |  |
| Type of graphics |  |  |  |  |
| Relevance of illustrations |  |  |  |  |
| List, tables, explained |  |  |  |  |
| Captions used for graphics |  |  |  |  |
| 1. **Layout and Typography** |  |  |  |  |
| Layout factors |  |  |  |  |
| Typography |  |  |  |  |
| Subheads (“chunking”) used |  |  |  |  |
| 1. **Learning stimulation, motivation** |  |  |  |  |
| Interaction used (question and answer format) |  |  |  |  |
| Behaviours are modelled and specific |  |  |  |  |
| Motivation – self efficacy |  |  |  |  |
| 1. **Cultural appropriateness** |  |  |  |  |
| Match in logic, language, experience |  |  |  |  |
| Cultural image and examples |  |  |  |  |

## Interactivity scale

**Active Control**

I felt that I had a lot of control over my visiting experiences at this Web site

While I was on the Web site, I could choose freely what I wanted to see

While surfing the Web site, I had full control over what I can do on the site*

While surfing the Web site, my actions decided the kind of experiences I got

**Two-Way Communication**

The Web site is effective in gathering visitors' feedback

This Web site facilitates two-way communication between the visitors and the site

It is easy to offer feedback to the Web site

The Web site makes me feel it wants to listen to its visitors

The Web site encourages visitors to talk back

The Web site gives visitors the opportunity to talk back

**Synchronicity**

The Web site processed my input very quickly

Getting information from the Web site is very fast

I was able to obtain the information I want without any delay

When I clicked on the links, I felt I was getting instantaneous information

The Web site was very fast in responding to my requests

### Scoring system for interactive features

| **1.** Is there an app associated with the website? | 0 = No 1 = Yes (This included searching play store, app store, and website)  If yes, what is the name of the app? |
| --- | --- |
| **2.** Does the website function the same on a phone screen and a laptop screen? (Mobile friendly) | 0 = No 1 = Yes. (This includes not having to scroll left and right to be able to view the content of the website; The whole website is fit to the dimensions of your phone |
| **3.** Does the website have a login? | 0 = No 1 = Yes. This includes typing in details (email etc) to be able to access website content |
| **4.** Does the website contain paid features? | 0 = No 1 = Yes. This includes (paying to access certain information, or promoting certain products for users to buy) |
| **5.** Is there a search function available for users to search for specific information? | 0 = No 1 = Yes |
| **6.**Is the page speed acceptable ? | 0 = No 1 = Yes |
| **7.**Is there a navigation menu? | 0 = No 1 = Yes (allow users to find what they’re looking for easily) |
| **8.**Does the website contain an interactive feature? | - Game - Video - Podcast - Chatbot - Q&A tab - Quizzes - Animation - Feedback form - Slide show - Rating - Frequently asked questions section (FAQ) - Language options🡪 if yes state what languages - Recipes - Read out loud - Social media links (twitter,FB) - Other |
| **9.**Do they address culture? | 0= No 1 = Yes  If yes, how? |
| **10.** Does the website have a Honcode certificate based on the 8 principles? | 0= No 1 = Yes |

## Scope, accuracy, and depth of information

| Evaluation tool | Number of criteria | Scoring system | Final composite score |
| --- | --- | --- | --- |
| REDCap tool based on the Australian Government’s guidelines on infant feeding, physical activity, and sleep | 65 subtopics | +1= Correct advice  -1= Incorrect  0= Not addressed  0.5=Partially addressed  “”=Not applicable | Excellent (≥90%)  Adequate (75%-89%)  Poor (≤74%) |
