# Supplementary material for: Interactivity, Quality, and Content of Websites Promoting Health Behaviors During Infancy: 6-Year Update of the Systematic Assessment
Source: J Med Internet Res. 2022 Oct 7;24(10):e38641. doi: 10.2196/38641 (PMC9587494; doi:10.2196/38641)
Supplement: Multimedia Appendix 2 [file jmir_v24i10e38641_app2.docx]

## Summary

| Targeted population | Websites with content targeted at parents of infants (≤1 year) |
| --- | --- |
| Websites | Websites aimed at providing parents with information/ resources on one of the following:   1. Promoting active play (tummy time, limit screen time etc.) 2. Appropriate milk feeding behaviors for the first year of life (breastfeeding, formula feeding, bottle feeding, feeding to appetite) 3. Appropriate solid food feeding behaviors (age of introduction, types of foods introduced, repeated exposure, varied exposure, reducing exposure to unhealthy food/beverages, feeding to appetite) 4. Infant sleeping behaviors |

## Website selection

The most widely used internet search engine (Google) will be used for the purpose of this study. The first 30 web pages that appear when entering key words such as infant feeding, breastfeeding, bottle feeding, formula feeding, baby food, solid foods, physical activity, screen time, tummy time, sleep in English will be selected if they meet the inclusion criteria.

### Selection criteria (Inclusion/ Exclusion)

| **Selection criteria** | **Include** | **Exclude** |
| --- | --- | --- |
| Website content | Any type of website that contains information on their page. If the information is on the website (even if it’s an article) include | - Electronic books, news, newspapers, websites that have a link that redirects you to an article, podcasts and downloadable word documents and pdfs |
| Intended audience | - Targets parents of children less than 1 year of age - English language, as primary language or language option | - Information only targeted at children ≥1 years - Non-English language |
| Variables of interest | Websites must report on at least one of the following:   - Healthy milk feeding behaviours:   *Breast, formula bottle, expressed breast milk, frequency, timing, correct preparation, feeding on demand, non-nutritive feeding*   - Healthy solid food feeding behaviours: *Age of introduction, types of foods introduced, repeated exposure, varied exposure, limit unhealthy food/beverages intake* - Promoting physical activity   *Tummy time, reducing restraint, allowing natural movement, child play time, reducing sedentary behaviours, screen time*   - Sleep   *Sleep schedule, sleep patterns, sleeping hours, sleep with bottle, co- sleep* | Exclude websites not reporting on at least one of the following topics:   - Infant physical activity   (*play time, tummy time, movement*)   - Milk feeding behaviours (*breastfeeding, formula feeding, frequency of feeding, frequency of pumping, and preparation of formula)* - Solid feeding behaviours (*first foods introduced, age of introduction, repeated exposure, limit unhealthy food and sugary beverages*). - Sleep behaviours   *Sleep schedule, sleep patterns, sleeping hours, sleep with bottle, co- sleep* |

### Search Terms

The following key terms were derived from “related searches” on Google and Facebook parent groups: Baby and toddler food recipes, Children nutrition and breastfeeding awareness, love my tummy time, formula fed babies Australia, Evidence Bases Feeding & Parenting: Formula, Breastfeeding & More, Baby Sleep Training Tips & Help.

Each key term will be screened and analysed by content separately.

| **General Key terms** |
| --- |
| SET 1: Healthy milk feeding behaviors |
| 1. Infant feeding |
| 1. Baby food |
| 1. Breast feeding |
| 1. Infant feeding to appetite |
| 1. Infant formula feeding |
| SET 2: Healthy solid food feeding behaviors |
| 1. Introducing solid foods to baby |
| 1. Good foods to start baby with no teeth |
| 1. Best puree for babies |
| 1. Solids and fussy babies |
| 1. Solids and milk feeding |
| SET 3: Promoting active play |
| 1. Infant active play |
| 1. Tummy time |
| 1. Screen time |
| SET 4: Sleep |
| 1. Infant sleep |
| 1. Baby co-sleep |

# Stage 1: Identification and selection of websites

Websites were be identified using the following approaches:

1. From safari access google

- Ensure that you have erased cookies and history from web browser history. This will avoid your previous search history influencing the search results.

2. Search each of the key terms from Set 1, 2, 3, or 4 individually (Table 3) in the search engine.

3. Record information of each search in RedCap, including:

- Key terms used
- Date of search conducted
- Website link
- Total number of results found
- Number of websites included, excluded, unsure on inclusion/exclusion, and justify

1. After identification of relevant websites for inclusion, proceed with assessment and evaluation of the websites using validated tools.

**Uncertain websites will be recorded and reviewed by another researchers. Discrepancies will be resolved by discussion.**

# Stage 2: Assessment and evaluation of information on the websites:

Validated tools are used to assess the quality, readability and suitability of web-based information on infant feeding practices, promoting healthy infant growth, infant sleep and infant movement.

Evidence based guidelines ‘Infant Feeding Guidelines’ (2012), ‘National Physical Activity Recommendations for Children 0-5 Years’ (2010), Community Paediatric Review (2015) and

Primary literature will be used to develop the criteria to assess accuracy and coverage/ completeness of the content of websites.

The following needs to be assessed from the selected websites:

1. Scope, accuracy, and depth of information – using criteria based on the Infant Feeding Guidelines, National Physical Activity Recommendations for Children 0-5 Years and primary literature
2. Website quality – using the Quality Component Scoring System (QCSS) tool and Health On the Net Code of Conduct (HONcode) tool
3. Interactivity- using a rating system is based on a standardised tool
4. Suitability of information – using the Suitability Assessment of Material (SAM)
5. Readability of information – using the Simple Measure of Gobbledygook (SMOG) and Flesch-Kincaid (F-K) tools and an overall sum of 6 readability tools ( SMOG, F-K, Flesch Reading Ease score, Gunning Fog, The Coleman-Liau Index, Automated Readability Index, Linsear Write Formula

Scope, accuracy, and depth of information

A scoring system will be used to measure the coverage the content derived from the Health-Related Website Evaluation Form (see appendix 1) , a tool used to assess the accuracy of health information websites. Coverage Scoring will be as follows: correct (+1), incorrect (-1), partially addressed (+0.5), absent (0) or not applicable.

The final coverage score given using the ratings from the Health-Related Website Evaluation Form, will be as follows: excellent (90% or higher), adequate (75-89%) or poor (less than 75%).

9 topics with 65 subtopics were identified using the National Health and Medical Research Council’s Infant Feeding Guidelines (2012), the Department of Health and Ageing’s National Physical Activity Recommendations for Children 0-5 Years (2010), Australian Family Physician (2015), and Community Paediatric Review (2015) (see appendix 1). These will be used to determine adequate coverage of the relevant material and the scientific accuracy of this information.

### Quality assessment

To evaluate the quality of the websites, validated tools including the Quality Component Scoring System (QCSS) tool will be used in this study (see appendix 3). The criteria of this tool includes: assess the quality of online information from the following 7 aspects: ownership, purpose, authorship, author qualification, attribution, interactivity, and currency. The Health-Related Website Evaluation Form (HRWEF) was also used for quality assessment. It consists of 30 items where each criterion is rated on a 3- point scale, scored as not applicable (0), disagree (1), or agree (2). It is divided into 7 main sections assessing the content, accuracy, author, currency, audience, navigation, and external links. An overall score was designated as excellent (90%-100%), adequate (75%-89%), or poor (0%-75%). Additionally, we will assess whether the websites have the Health on the Net Code of Conduct (HONcode) certification.

### Suitability of information

The SAM was used to objectively assess the suitability of the information (see appendix 1). The SAM tool is assessed using various areas of the website including: content, literacy level, graphics, layout, interaction with readers, learning stimulation and motivation and cultural appropriateness.

A scoring system of superior (+2), adequate (+1), not suitable (0) or not applicable, is reported based on the objective criteria of the instrument.

### Readability of information

The two most commonly used tools include F-K [7] and SMOG test, was used in this evaluation. Additionally, an overall sum of 6 readability tools ( SMOG, F-K, Flesch Reading Ease score, Gunning Fog, The Coleman-Liau Index, Automated Readability Index, Linsear Write Formula will be used.
