# Supplementary material for: Interactivity, Quality, and Content of Websites Promoting Health Behaviors During Infancy: 6-Year Update of the Systematic Assessment
Source: J Med Internet Res. 2022 Oct 7;24(10):e38641. doi: 10.2196/38641 (PMC9587494; doi:10.2196/38641)
Supplement: Multimedia Appendix 3 [file jmir_v24i10e38641_app3.docx]

## Appendix 3

### Summary score for all included websites

|  | Content score (%) | HRWEF (%) | QCSS (%) | SAM (%) | Interactivity scale (%) |
| --- | --- | --- | --- | --- | --- |
| Nemours Kids Health | 61 | 91 | 77 | 84 | 73 |
| Stanford children's health | 80 | 78 | 62 | 50 | 67 |
| Healthline | 70 | 89 | 85 | 45 | 47 |
| MAYO CLINIC | 68 | 91 | 77 | 61 | 50 |
| Tresillian | 38 | 74 | 38 | 50 | 33 |
| Australian Breastfeeding Association | 74 | 95 | 77 | 60 | 87 |
| Grow by WebMD | 52 | 85 | 77 | 55 | 37 |
| Healthy Children | 64 | 87 | 77 | 63 | 63 |
| Queensland Health | 73 | 91 | 77 | 58 | 53 |
| Centers for Disease Control and Prevention | 63 | 86 | 69 | 58 | 63 |
| Cleveland Clinic | 50 | 86 | 77 | 53 | 53 |
| Raising children network | 69 | 96 | 77 | 93 | 87 |
| Nutricia | 38 | 90 | 62 | 63 | 77 |
| What to expect | 58 | 89 | 85 | 67 | 70 |
| Baby Center | 76 | 93 | 92 | 45 | 67 |
| Nestle mum and me | 58 | 77 | 31 | 50 | 70 |
| Newborn baby | 52 | 82 | 31 | 41 | 63 |
| Pregnancy Birth & Baby | 77 | 91 | 69 | 53 | 70 |
| Better health channel | 72 | 86 | 69 | 55 | 40 |
| Karitane | 42 | 89 | 62 | 56 | 73 |
| Medela | 60 | 82 | 54 | 53 | 57 |
| Government of Western Australia | 57 | 86 | 54 | 48 | 67 |
| Baby care advice | 45 | 86 | 69 | 58 | 63 |
| Kelly mom | 54 | 91 | 77 | 45 | 50 |
| USDA | 50 | 89 | 31 | 65 | 60 |
| Very well family | 71 | 91 | 77 | 65 | 67 |
| Haberman baby | 27 | 84 | 46 | 33 | 77 |
| Heinz | 77 | 84 | 23 | 44 | 70 |
| Victoria state government | 25 | 86 | 69 | 38 | 43 |
| NHS | 63 | 87 | 54 | 45 | 70 |
| Mott Children’s Hospital | 37 | 81 | 54 | 35 | 30 |
| Ngala raising happiness | 34 | 84 | 38 | 48 | 63 |
| a2 nutrition | 50 | 86 | 46 | 58 | 77 |
| Tommy's | 55 | 87 | 69 | 60 | 47 |
| First Five Years | 38 | 83 | 46 | 43 | 47 |
| Eat right academy of nutrition and dietetics | 38 | 78 | 62 | 50 | 37 |
| Happy family organics | 68 | 87 | 77 | 63 | 73 |
| PBC expo | 59 | 82 | 31 | 53 | 47 |
| Colorado state university | 18 | 76 | 46 | 35 | 40 |
| Parents line | 69 | 80 | 46 | 48 | 70 |
| My little moppet | 41 | 87 | 62 | 61 | 47 |
| Parenting first cry | 41 | 74 | 62 | 35 | 20 |
| New ways nutrition | 44 | 83 | 69 | 65 | 43 |
| The bump | 80 | 83 | 46 | 55 | 40 |
| Hello motherhood | 63 | 84 | 46 | 40 | 30 |
| Bellamys organic | 41 | 74 | 92 | 53 | 47 |
| Made for mums | 55 | 80 | 46 | 48 | 43 |
| One handed cook | 75 | 91 | 54 | 60 | 47 |
| Creative nourish | 58 | 87 | 77 | 53 | 67 |
| Wholesome baby food | 25 | 89 | 23 | 38 | 37 |
| Annabel Karmel | 35 | 86 | 62 | 56 | 47 |
| Baby sleep site | 42 | 86 | 69 | 42 | 70 |
| Parenting science | 36 | 89 | 77 | 33 | 47 |
| Solids starts | 57 | 89 | 69 | 53 | 53 |
| Plunket | 71 | 91 | 62 | 69 | 87 |
| Plum organics | 26 | 84 | 31 | 36 | 47 |
| Bubs Australia | 44 | 80 | 23 | 58 | 50 |
| Play group NSW | 36 | 82 | 62 | 50 | 47 |
| Dr Golly | 22 | 84 | 77 | 42 | 47 |
| Pathways | 55 | 89 | 54 | 52 | 37 |
| Screen time | -13 | 61 | 54 | 19 | 27 |
| Sleep foundation | 67 | 95 | 85 | 50 | 33 |
| let's sleep | 57 | 82 | 62 | 44 | 70 |
| Red nose | 66 | 87 | 62 | 43 | 47 |
| Basis | 50 | 77 | 23 | 38 | 23 |
| Little sparkles | 64 | 93 | 69 | 47 | 33 |
