# Supplementary material for: Interactivity, Quality, and Content of Websites Promoting Health Behaviors During Infancy: 6-Year Update of the Systematic Assessment
Source: J Med Internet Res. 2022 Oct 7;24(10):e38641. doi: 10.2196/38641 (PMC9587494; doi:10.2196/38641)

## Appendix 4

**Health-Related Website Evaluation Form (HRWEF)**

| HRWEF Rating | n of websites | Percentage score (%) |
| --- | --- | --- |
| Excellent (at least 90%) | 13 | 20% |
| Adequate (at least 75%) | 49 | 72% |
| Poor (<75%) | 4 | 6% |

#### **The Quality Component Scoring System (QCSS)**


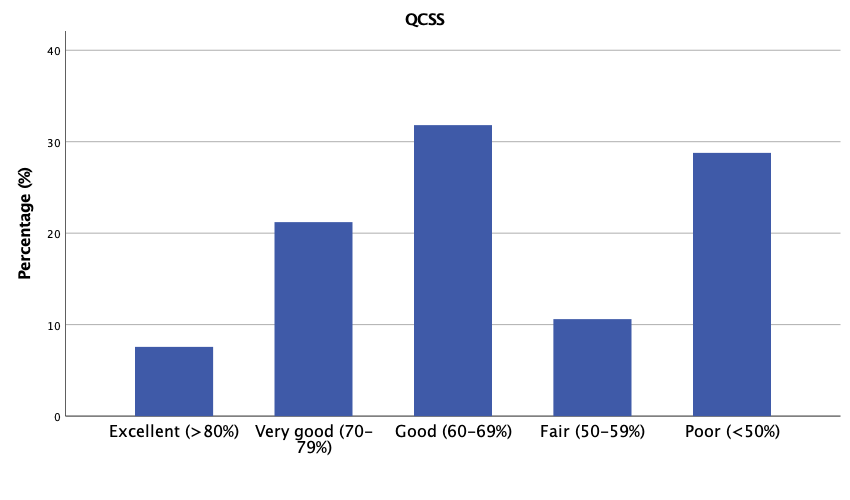


**Readability**


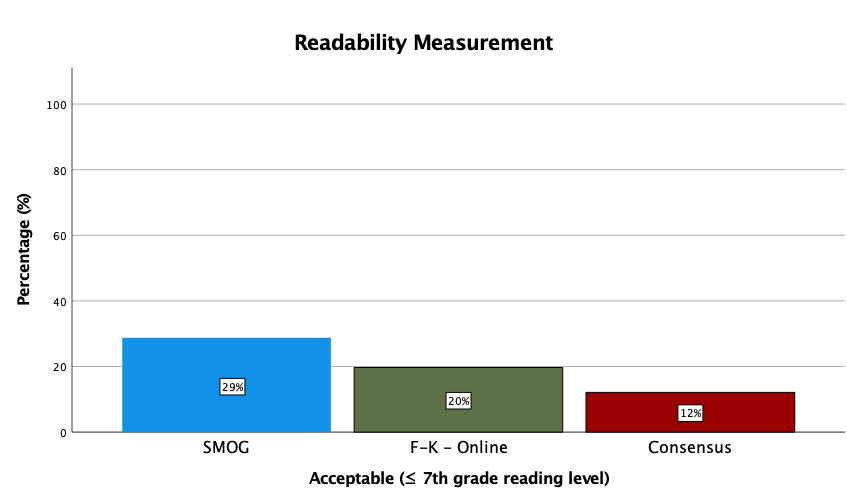


**Website Interactivity Table**

|  | Disagree  n(%) | Partially agree  n(%) | Agree  n(%) |
| --- | --- | --- | --- |
| **Active Control** | | | |
| I felt that I had a lot of control over my visiting experiences at this Web site | 2(3%) | 47(71%) | 17(26%) |
| While I was on the Web site, I could choose freely what I wanted to see | 2(3%) | 41(62%) | 23(35%) |
| While surfing the Web site, I had full control over what I can do on the site | 4(6%) | 41(62%) | 21(32%) |
| While surfing the Web site, my actions decided the kind of experiences I got | 1(2%) | 41(62%) | 24(36%) |
| **Two-Way Communication** | | | |
| The Web site is effective in gathering visitors' feedback | 15(23%) | 45(68%) | 6(9%) |
| This Web site facilitates two-way communication between the visitors and the site | 53(80%) | 6(9%) | 7(10%) |
| It is easy to offer feedback to the Web site | 17(26%) | 36(55%) | 13(20%) |
| The Web site makes me feel it wants to listen to its visitors | 12(18%) | 45(68%) | 9(14%) |
| The Web site encourages visitors to talk back | 11(17%) | 47(71%) | 8(12%) |
| The Web site gives visitors the opportunity to talk back | 5(8%) | 43(65%) | 18(27%) |
| **Synchronicity** | | | |
| The Web site processed my input very quickly | 3(5%) | 47(71%) | 16(24%) |
| Getting information from the Web site is very fast | 6(9%) | 45(68%) | 15(23%) |
| I was able to obtain the information I want without any delay | 8(12%) | 48(73%) | 10(15%) |
| When I clicked on the links, I felt I was getting instantaneous information | 4(6%) | 36(55%) | 26(39%) |
| The Web site was very fast in responding to my requests | 3(5%) | 45(68%) | 18(27%) |

**Website Interactivity Graph**


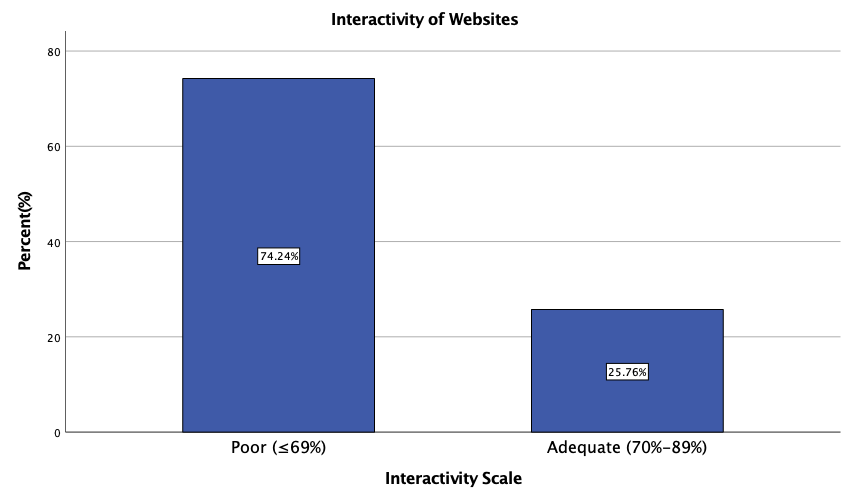

Supplement: Multimedia Appendix 4 [file jmir_v24i10e38641_app4.docx]
